# Drug repurposing in alternative medicine: herbal digestive Sochehwan exerts multifaceted effects against metabolic syndrome

**DOI:** 10.1038/s41598-019-45099-x

**Published:** 2019-06-21

**Authors:** Dong-Woo Lim, Hyuck Kim, Young-Mi Kim, Young-Won Chin, Won-Hwan Park, Jai-Eun Kim

**Affiliations:** 1 Department of Pathology, College of Korean Medicine, Dongguk University, Goyang, 10326 Republic of Korea; 2 Department of Diagnostics, College of Korean Medicine, Dongguk University, Goyang, 10326 Republic of Korea; 3 College of Pharmacy and Integrated Research Institute for Drug Development, Dongguk University, Goyang, 10326 Republic of Korea

**Keywords:** Drug discovery, Drug discovery, Metabolic disorders, Metabolic disorders, Therapeutics

## Abstract

New drug development is a challenging process that requires high-risk, huge costs and long lead times. Therefore, drug repurposing is considered a strategic and economic way towards successful drug development. Sochehwan (SCH) is a herbal formula well known as a digestive aid in traditional oriental medicine, is referred to in classic medical texts, and is available as an over-the-counter drug for indications of digestive ailments. Interestingly, another medical text written in earlier age describes different indication of SCH yet to be examined. We conducted a series of investigations using maturated adipocytes, free fatty acid (FFA) induced hepatic steatosis model *in vitro* and high-fat diet (HFD) mice model *in vivo*. Exposure to SCH regulated expression of adipogenic genes and proteins, significantly inhibiting formation of lipid droplets in 3T3-L1 cells. Similarly, SCH treatment modulated proteins related with energy metabolism decreasing lipid accumulation in FFA induced HepG2 cells. Furthermore, HFD-fed c57BL/6 J mice supplemented with SCH exhibited significant changes in serum glucose and lipid profiles. Histologic analysis of mice liver and adipose tissue showed that SCH administration attenuated hepatic steatosis and hypertrophy of adipose tissue. In overall, the results show that SCH can potentially be used to treat metabolic syndrome (MetS) by enhancing glucose metabolism and inhibiting lipogenesis through activating AMP-activated protein kinase (AMPK) and its downstream signaling. Furthermore, it seems to be a feasible drug repurposing strategy for drugs originating from alternative medicine to revise the value for buried indications of some herbal prescription in old traditional Chinese Medicine (TCM) classics.

## Introduction

Drug development is a challenging process that requires huge cost and long-term investment with high-risk of failure in trials^1^. To reduce time-to-market costs and risk, alternative approaches were considered by investigators^2–5^. One type of drug repurposing (also called drug repositioning) refers to the development of known drugs for new indications^6^. Based on well-established preliminary data (clinical safety data with organized reports of adverse effects, manufacturing methods and, pharmacokinetics (ADME)), drug repurposing is regarded as a cost-effective and time-saving route to success as compared to novel drug development involving high-throughput screening (HTS)^7^.

Herbal medicines used to treat diseases for centuries have debates over their efficacy and safety in the clinic^8^. Based on original efficacy of usage for certain indications in texts, some prominent herbal prescriptions have been developed into novel drugs^9^. Examples of approved natural product drugs in Korea include Layla Tab^10^. and Joins Tab^11^. which are used for osteoarthritis and which derive from herbal prescriptions from Korean medicine. In addition, there is the high possibility of seeking new indications for existing herbal prescriptions which can lead to new drug development with drug repurposing strategies. Some reported cases support the theory. The author previously reported that Samjunghwan, a herbal prescription in Korean medicine, referred to as an anti-aging agent in original text^12^, has notable effects on hyperlipidemia^13^.

Metabolic syndrome (MetS) is a complex set of metabolic abnormalities including hypertension, abdominal obesity, insulin resistance, dyslipidemia, and diabetes^14^. Striking increases in the incidence of MetS is now regarded as a new epidemic threatening human health worldwide^15^. MetS is highlighted as a baseline status for lethal diseases characterized by systemically deteriorated energy metabolism^16^, abnormal immune response^17^, and dysregulation of endocrine function^18^, eventually leading to increased mortality^19^.

Causes of metabolic syndrome cannot be simply defined, as both environmental factors as well as genetic factors are important in its etiology^20^. However, it is insufficient to explain the explosive growth in MetS as due to genetic factors, but rather it would be reasonable to attribute its occurrence to rapid changes in lifestyle, as it is largely preventable with lifestyle modifications^21^.

Various agents targeting normalization of individual parameters in metabolic syndrome are already used in the clinical field or under development. Available treatments for metabolic syndrome include lipid-lowering agents^22^, insulin sensitizers and weight loss medications^23^. Still, there is a need to find natural compounds which are side effect free, and work in multi-effective ways.

**Figure 1:**
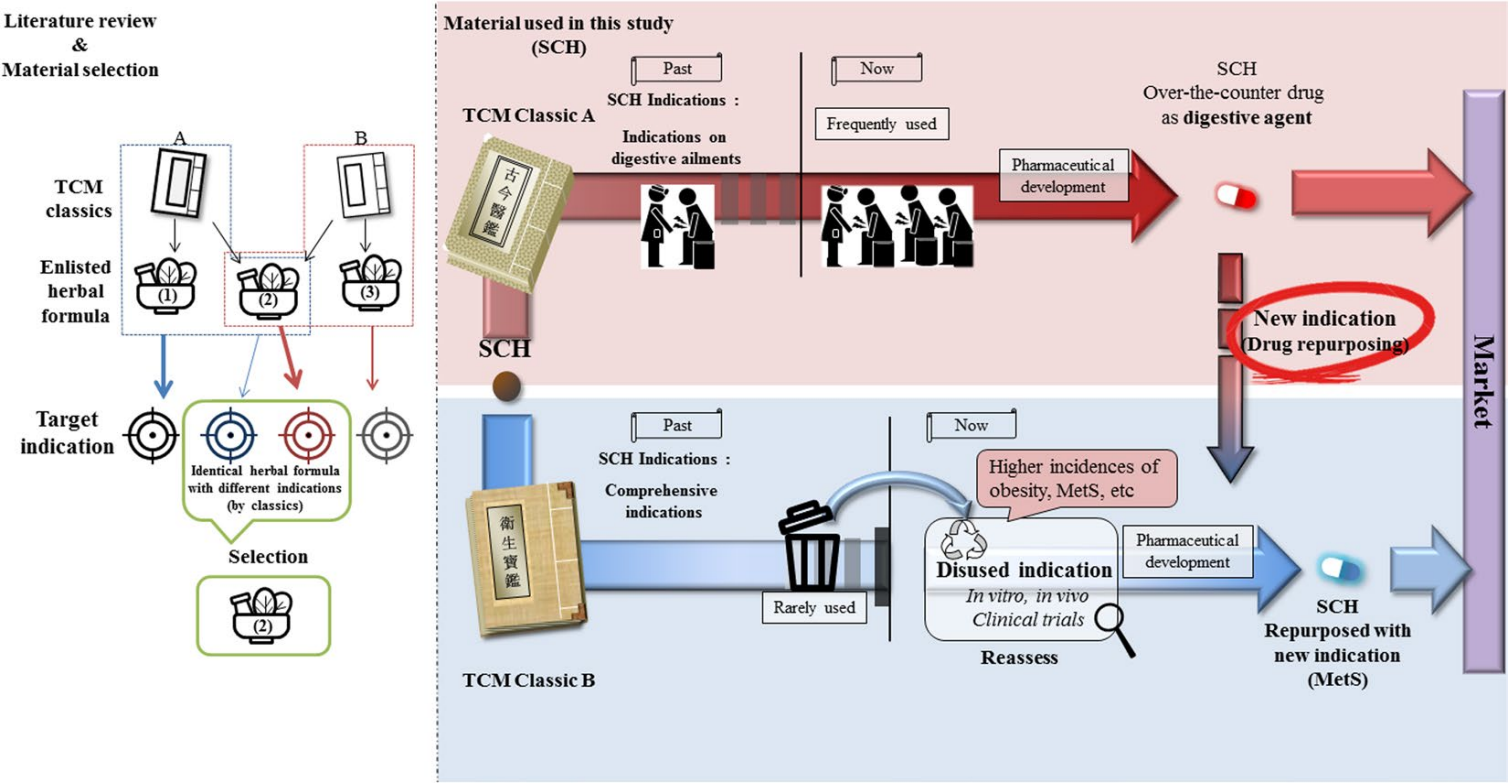
Schematic diagram describing the concept of literature-based drug repositioning of herbal prescription from two different TCM classics applied in this study.

Sochehwan (SCH), a herbal formula of traditional Korean Medicine, is widely used to treat digestive ailments including abdominal pain, dyspepsia, abdominal fullness and distention^24^. The formula consists of three herbs; *Pharbitis Semen* (*Pharbitis nil* Choisy), *Trogopterori Faeces* (*Faeces of Trogopterus xanthipes*), *Cyperi Rhizoma* (*Cyperus rotundus* L.). In a previous study, SCH recovered gastric mucosal barrier damage in mice with reduced NF-κB p-65, iNOS and COX-2 levels^24^. The prescription has been developed and commercialized by several pharmaceutical companies as an over-the-counter drug in Korea. These products are indicated for digestive ailments based on an authoritative and well-known original medical text (Gogeumeuigam)^25^. However, interestingly, there is another classic medical text (Wisaengbogam)^26^ which describes different indications for SCH with the same herbal composition (Fig. 1). To date, limited empirical study has been conducted to examine efficacy of SCH on new indications including non-alcoholic fatty liver disease (NAFLD)^27^. We expected that SCH may have other indications besides well-known digestive ailments according to another medical literature of TCM.

We investigated the effect of SCH on various parameters of metabolic syndrome through three different models. Modulating effects of SCH on lipid accumulation and glucose utilization were assessed in a hepatic steatosis model. Also, regulatory impacts of SCH on preadipocyte differentiation and lipid biosynthesis were studied in adipocytes. Moreover, the effects of SCH administration on parameters of metabolic syndrome of high fat diet (HFD)-fed mice were evaluated. In conclusion, through a series of investigations, we demonstrated efficacy of SCH on metabolic syndrome to suggest the possibility of new drug development through a drug repurposing process.

## Results

### Effect of SCH on 3T3-L1 and HepG2 cell viability

Cytotoxicity of SCH on 3T3-L1 preadipocyte cells was determined prior to starting a 3T3-L1 study. Incubation of 3T3-L1 cells with SCH for 24 h did not exhibit any evident cytotoxicity under concentrations of 50 μg/ml which showed 102.2% viability compared with non-treated controls. However, viability decreased to 93.1% at concentration of 75 μg/ml. For prolonged SCH treatment on 3T3-L1 cells, a concentration of 50 μg/ml was considered maximal (Fig. S1A).

Incubation of HepG2 cells with SCH for 24 h did not show any notable cytotoxicity under a concentration of 50 μg/ml (>90% viability). According to these results, a concentration of 50 μg/ml was deduced as the maximal concentration in further studies with HepG2 cells (Fig. S1B).

### SCH inhibited preadipocyte differentiation in 3T3-L1 cells

**Figure 2:**
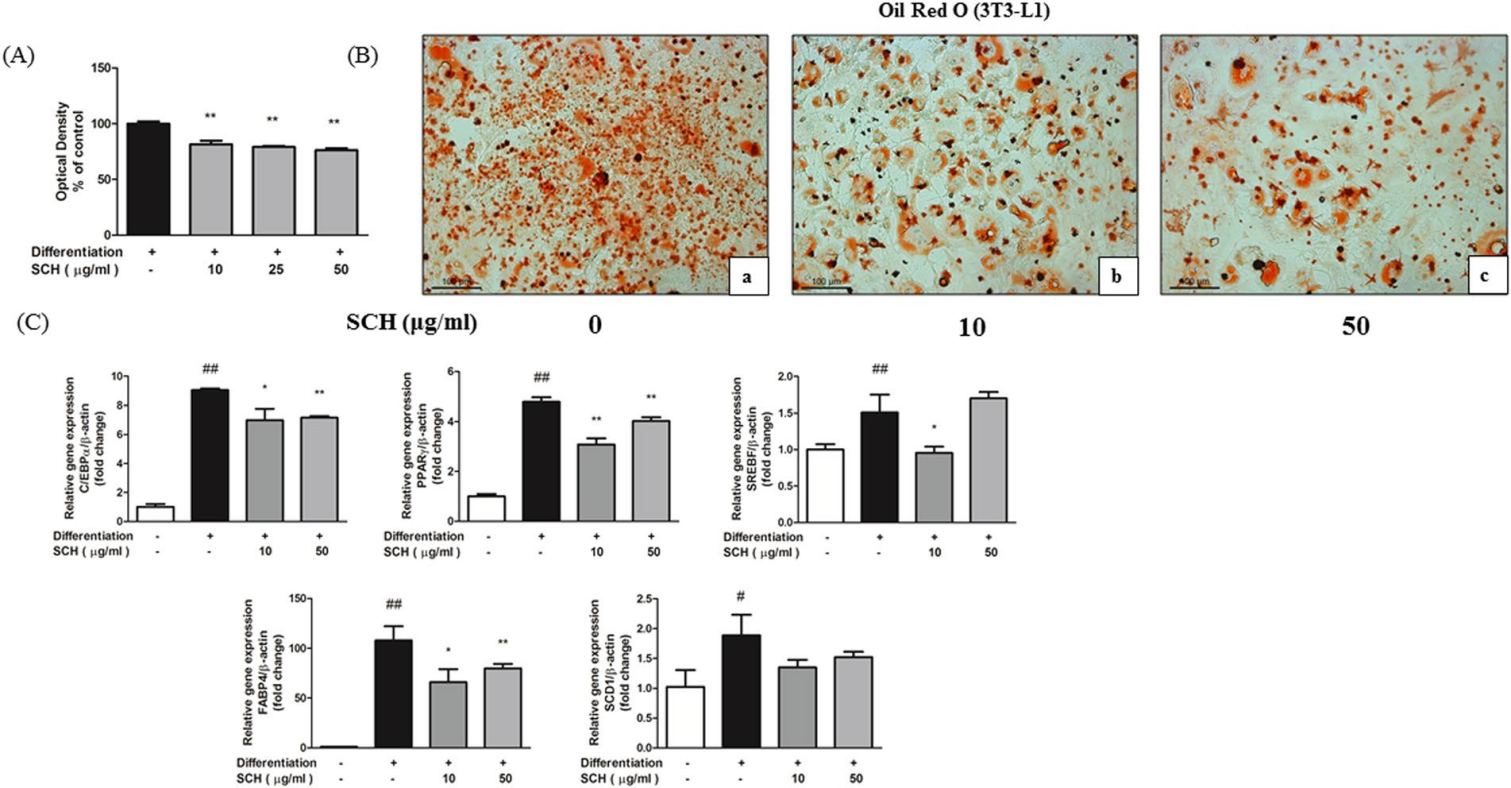
Effect of SCH on 3T3-L1 preadipocyte differentiation and lipogenesis. Measurement of relative lipid accumulation in maturated 3T3-L1 cells with spectrophotometric measurement of Oil Red O staining. Intracellular lipid accumulation and adipocyte maturity were investigated with microscopic analysis (**B**). Cells were untreated (a), treated with 10 μg/ml SCH (b) or treated with 50 μg/ml SCH (c). Images were taken at magnification of 200×. Quantitative real-time PCR showing the effect of SCH on gene expression involved in adipocyte differentiation and lipogenesis (**C**). All gene expression was normalized to β-actin gene expression to calculate relative expression. Data are expressed as mean ± SD.

The effect of long-term SCH co-treatment on preadipocyte differentiation was investigated in differentiating 3T3-L1 cells. Oil Red O staining after 13 days of SCH treatment showed by optical density and microscopic image (Fig. 2A,B) that treatment with SCH caused significantly inhibited accumulation of intracellular lipid contents in 3T3-L1 cells by preventing differentiation. Re-dissolved in pure isopropanol, Oil Red O stain was significantly reduced by SCH treatment at concentrations of 10, 25, 50 μg/ml. Fewer lipid-laden differentiated 3T3-L1 cells were observed in SCH treated groups as compared to non-treated groups.

### SCH regulated adipogenic gene expression in maturated 3T3-L1 cells

Effect of SCH on expression of genes involved in adipogenesis was investigated by real-time quantitative PCR. Adipogenic markers including CCAAT/Enhancer Binding Protein alpha (C/EBPα), Peroxisome proliferator-activated receptor gamma (PPARγ), Stearoyl-CoA desaturase 1 (SCD1), Sterol Regulatory Element Binding Factor (SREBF), Fatty Acid Binding Protein 4 (FABP4) were all notably increased by differentiation (Fig. 2C). However, exposure to SCH significantly decreased the gene expression of C/EBPα, PPARγ, SREBF, FABP4 (except with SREBF at 50 μg/ml SCH concentration) in maturated 3T3-L1 cells. SCD1 gene expression was decreased by SCH treatment but the difference was not significant.

### SCH regulated proteins involved in energy metabolism in maturated 3T3-L1 Cells

**Figure 3:**
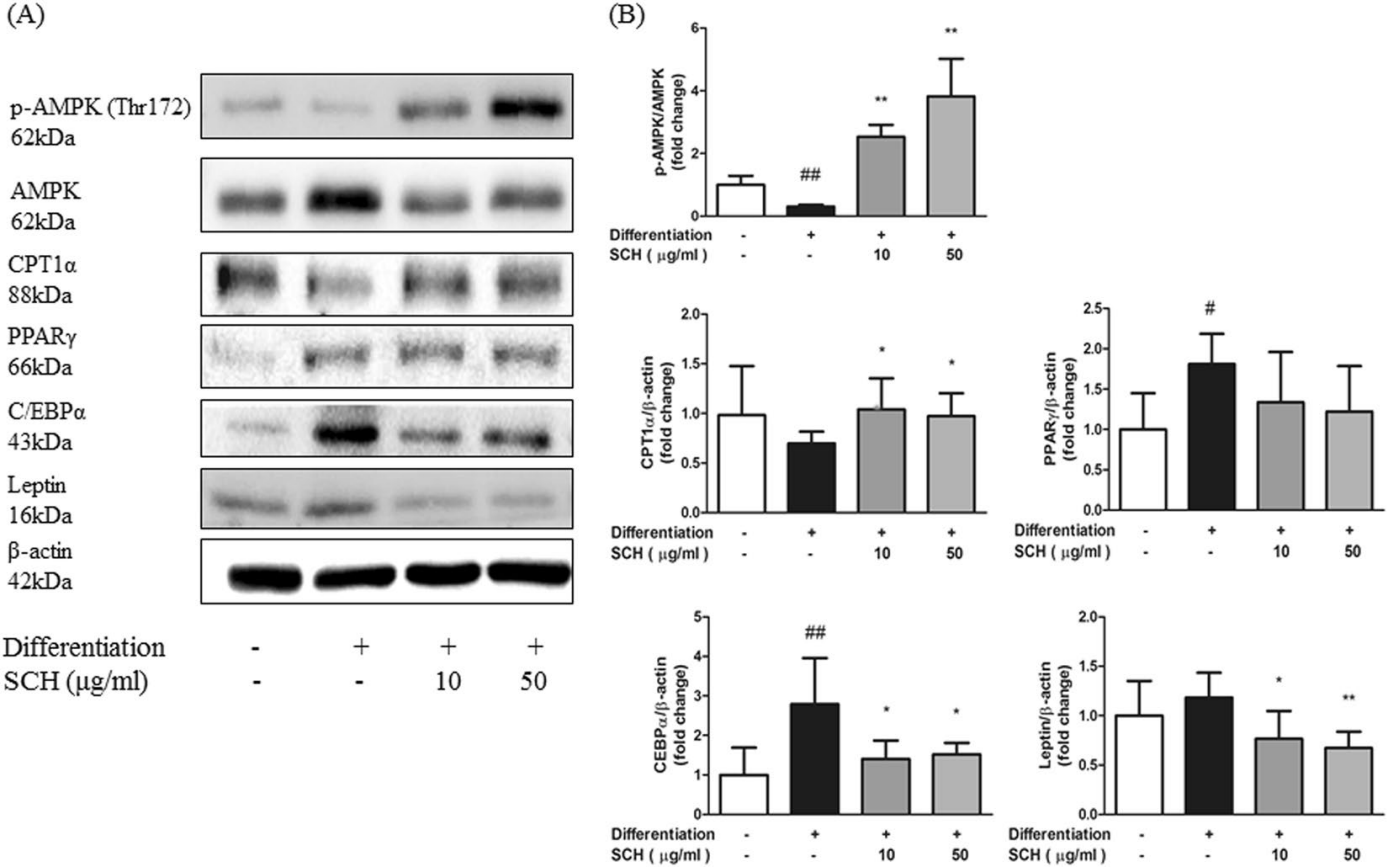
Western blot analysis showing the effect of SCH on phosphorylation of AMPK or protein expression related to energy metabolism and lipogenesis in maturated 3T3-L1 adipocytes. (**A**) Representative blots of proteins are depicted. (**B**) Band intensity was measured with densitometric analysis and normalized to the intensity of non-phosphorylated AMPK or β-actin. Data are expressed as mean ± SD.

Densitometry of immunoblot images revealed effects of SCH on protein levels involved in preadipocyte differentiation and adipogenesis. Expression of phosphorylated AMP-activated protein kinase (AMPK) level in 3T3 cells was increased by SCH at both concentrations tested (10, 50 μg/ml) (Fig. 3A,B).

Carnitine palmitoyltransferase 1 alpha (CPT1α) protein expression was significantly increased in 3T3-L1 cells by SCH at a concentration of 50 μg/ml as compared to the non-treated differentiated group. PPARγ protein level was slightly elevated by differentiation and further decreased by SCH but differences were not significant. Meanwhile, C/EBPα protein level was markedly increased by differentiation but reduced by SCH treatment at both concentrations. Expression levels of leptin were significantly decreased by SCH treatment in maturated adipocytes (Fig. 3A,B).

### SCH attenuated hepatic steatosis induced by FFA in HepG2 cells

**Figure 4:**
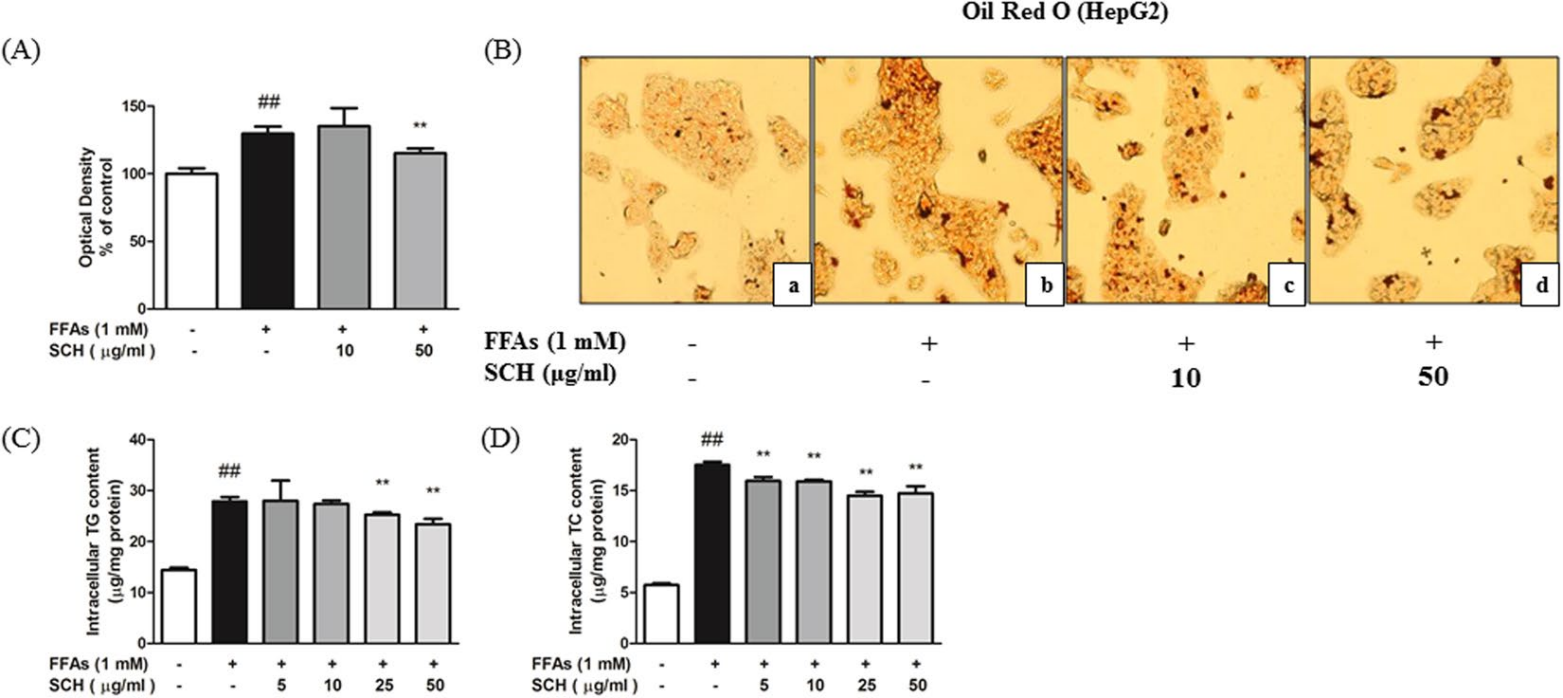
Effect of SCH on lipid accumulation in FFA-induced hepatic steatosis HepG2 model. (**A**) Measurement of relative lipid accumulation in FFA-induced steatotic HepG2 cells with spectrophotometric measurement of Oil Red O staining. Intracellular lipid accumulation was observed by microscopic analysis (**B**). Cells were untreated (a), FFA-induced (b), exposed to FFA with 10 μg/ml of SCH (c) or exposed to FFA with 50 μg/ml SCH (d). Images were taken at magnification of 200×. Impact of SCH on intracellular triglycerides levels (**C**) and total cholesterol levels. Data are expressed as mean ± SD.

The effect of SCH co-treatment on lipid accumulation was investigated in the FFA-induced HepG2 steatosis model. Results of Oil Red O staining showed that treatment with 1 mM FFA caused a significant increase of intracellular lipid contents in HepG2 cells, demonstrated by absorbance in 520 nm (Fig. 4A). Oil Red O stained, lipid droplets in cells were more evident in the FFA treated group compared to the untreated group (Fig. 4B). In addition, there was significant augmentation in intracellular triglycerides and total cholesterol by FFA treatment (Fig. 4C,D). However, there were significant declines in the absorbance of Oil Red O staining (50 μg/ml), triglycerides (25, 50 μg/ml), and total cholesterol (5, 10, 25, 50 μg/ml) by 48 h SCH treatment.

### SCH attenuated impaired glucose uptake rate caused by FFA exposure

**Figure 5:**
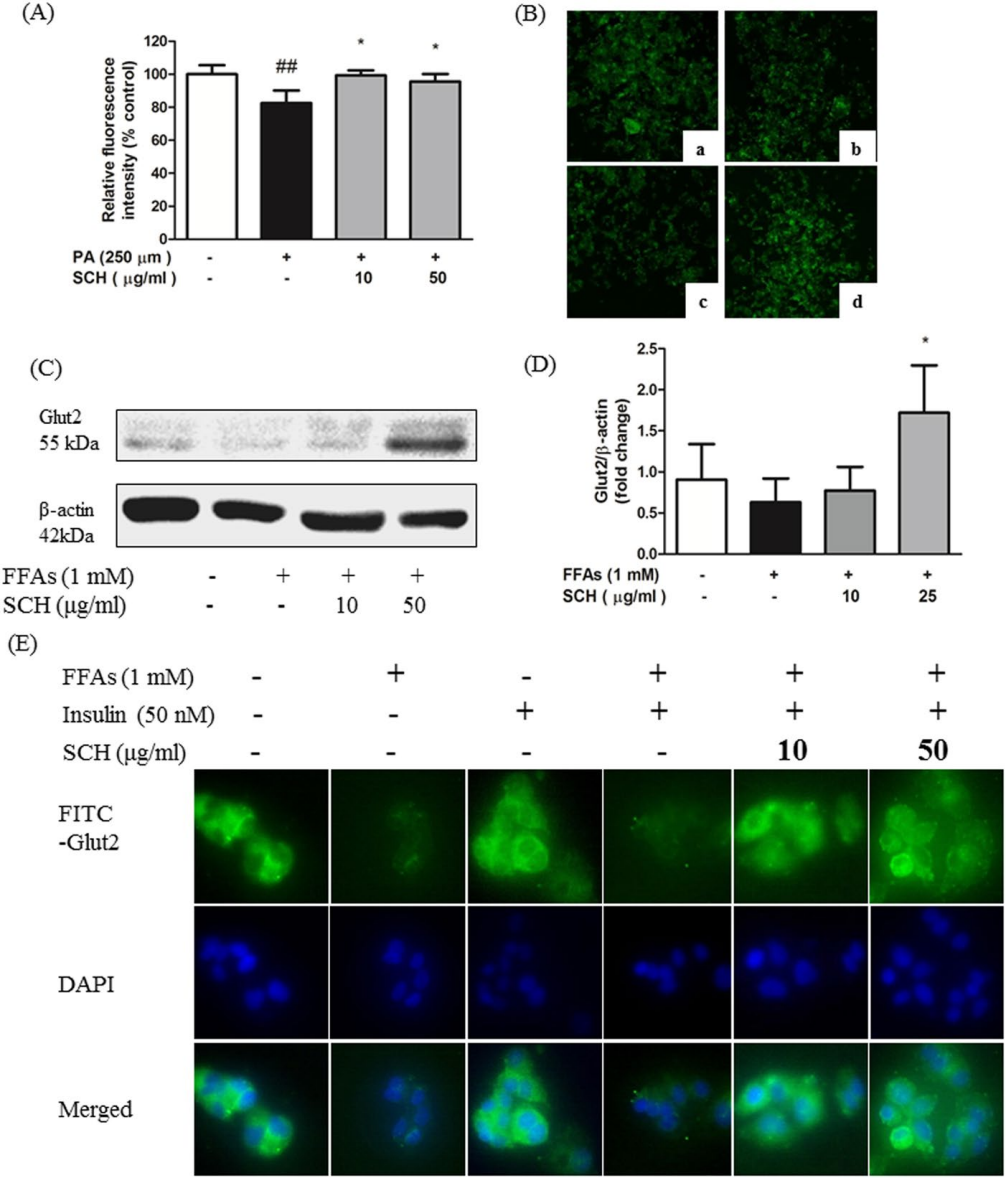
Impacts of SCH on glucose uptake and GLUT2 protein in FFA-induced steatotic HepG2 cells. (**A**) Relative fluorescence intensity (2-NBDG) of HepG2 cells implying glucose uptake rate. (**B**) Representative fluorescence images of steatotic HepG2 showing relative glucose uptake rate. Cells were untreated (a), FFA-treated (b), exposed to FFA with 10 μg/ml of SCH (c), exposed to 50 μg/ml of SCH (d). (**C**) Representative blot of GLUT2 protein is depicted. (**D**) Band intensity was measured with densitometric analysis and normalized to the intensity of β-actin. (**E**) Immunofluorescence images showing the impact of SCH on localization of Glut 2 protein in insulin-stimulated FFA-induced HepG2 cells. Images were taken at magnification of 400×. Data are expressed as mean ± SD.

The effect of SCH treatment on glucose uptake was investigated in FFA-treated HepG2 cells. Relative fluorescence measurements revealed that 250 μM palmitic acid notably diminished glucose utilization in HepG2 cells as compared to untreated cells. However, SCH treatment at 10, 50 μg/ml concentration significantly elevated glucose uptake (Fig. 5A). Fluorescent microscopic images showed similar results with fluorescence intensity (Fig. 5B).

### SCH improved glucose facilitation by GLUT2 externalization

The expression level and localization of GLUT2 protein, as a predominant glucose transporter in hepatocytes, was traced using an immunofluorescence technique. Quantitative analysis of Western blot images showed significant increases in GLUT2 levels in steatotic HepG2 cells exposed to SCH treatment (Fig. 5C,D). Without permeabilization process in advance of immunofluorescence staining, antibodies conjugate only with externalized proteins. As shown, translocation of GLUT2 protein to the plasma membrane was inhibited in HepG2 cells regardless of the presence of insulin stimulation by 1 mM FFA treatment. However, SCH treatment augmented externalization of GLUT2 protein and this was demonstrated by increased fluorescence intensity at both concentrations (10, 50 μg/ml) (Fig. 5E).

### SCH regulated proteins involved in energy metabolism in HepG2 Cells

**Figure 6:**
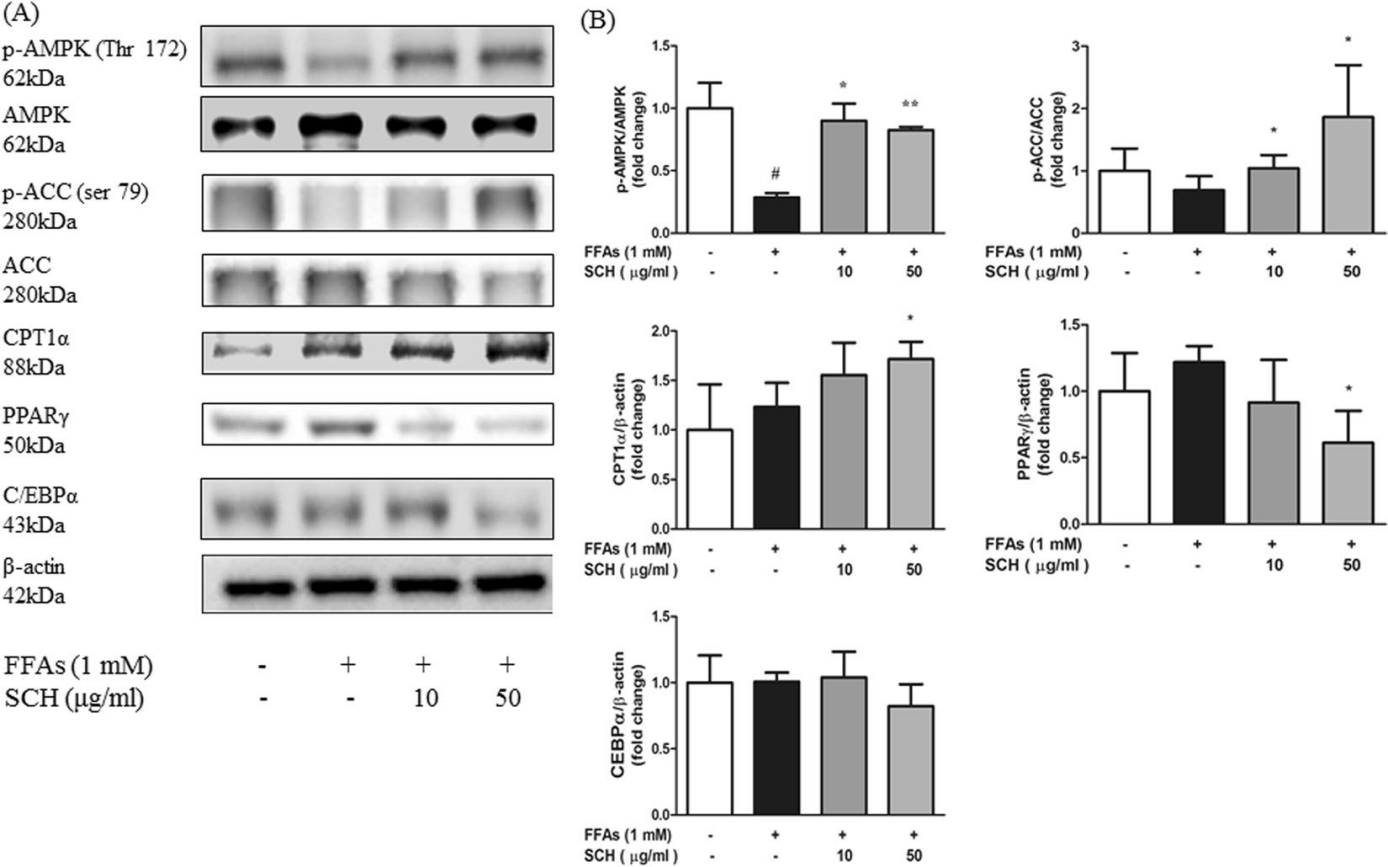
Western blot analysis showing the effect of SCH on phosphorylation of AMPK, ACC or protein expression related to energy metabolism and lipogenesis in FFA-induced steatotic HepG2 cells. (**A**) Representative blots of proteins are depicted. (**B**) Band intensity was measured with densitometric analysis and normalized to the intensity of non-phosphorylated AMPK or non-phosphorylated ACC or β-actin. Data are expressed as mean ± SD.

Immunoblot results from steatotic HepG2 cells indicated that a decrease in phosphorylated AMPK levels was induced by FFA treatment. However, phosphorylation was significantly elevated by SCH treatment compared to that of the normal group at both concentrations (10, 50 μg/ml). Phosphorylated Acetyl-CoA carboxylase (ACC) level was slightly decreased by FFA treatment but notably increased by SCH treatment at both concentrations (Fig. 6A,B).

Moreover there was a significant increase in CPT1α expression in HepG2 cells by SCH at a concentration of 50 μg/ml. Expression of PPARγ was slightly elevated by FFA treatment, however, it was markedly decreased by 50 μg/ml of SCH. Expression of C/EBPα was insignificantly reduced (Fig. 6A,B).

### SCH alleviated insulin resistance in FFA-induced steatotic HepG2 Cells

Immunoblots from insulin-stimulated steatotic HepG2 cells showed that exposure to FFA notably decreased the ratio of phosphorylated/non-phosphorylated protein kinase B (AKT) and phosphoinositide 3-kinase (PI3k) levels (Fig. S2A,B). However, immunoblots of steatotic cells co-treated with SCH showed increased phosphorylation levels of AKT as compare to non-treated steatotic cells at the higher dose (50 μg/ml). Accordingly, phosphorylated PI3k levels were increased by SCH co-treatment in dose-dependent manner, but not significantly.

### Multi-regulatory effects of SCH on lipid metabolism

The hierarchical clustergram heatmap represents relative expression levels for three groups (non treated, FFA-treated, and FFA-cotreated with SCH) on genes involved in human steatosis (genes from RT^2^ profiler human fatty liver PCR array including insulin signaling pathway, adipokine signaling pathway, NIDDM, metabolic pathway, inflammatory responses, apoptosis) (Fig. S3). Eighty-four genes related to human fatty liver were analyzed with PCR array simultaneously. The results showed a striking regulatory effect of SCH on various gene expressions. Clustering of non-treated group is separated from FFA-treated group and close to the SCH group. Among genes analyzed, CPT1A, pyruvate dehydrogenase lipoamide kinase isozyme 4 (PDK4) and interleukin-10 (IL-10) gene expressions were notably increased (1.8, 1.9, 1.8-fold) in SCH treated group as compared to FFA treated group, recovering its expression levels to the normal levels seen in the untreated group. Contrastingly, lipoprotein lipase (LPL) and suppressor of cytokine signaling (SOCS3) gene expressions were decreased (to 0.36 and 0.2-fold) by SCH treatment.

The result of this PCR array implies that SCH treatment has the potential to reconstitute clustered gene expression similar to normal conditions in steatotic HepG2 cells.

### SCH regulated glucose homeostasis and serum lipid levels in HFD-fed mice

**Figure 7:**
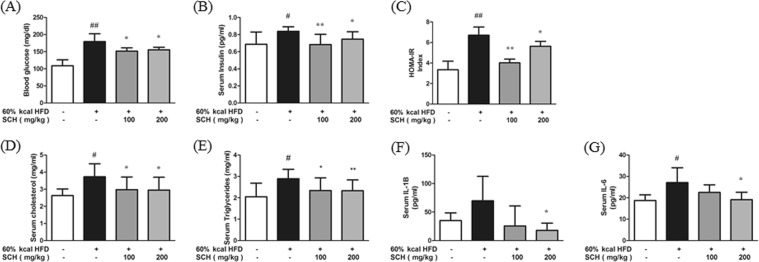
Effect of 15 week SCH administration on blood glucose homeostasis, serum lipid levels, and inflammatory cytokine levels of HFD-fed c57BL/6 mice. Fasting blood glucose levels (**A**) and fasting serum insulin levels (**B**) were measured after 12 h starvation. (**C**) HOMA-IR index was calculated by multiplying each individual’s fasting glucose levels with insulin levels. Serum cholesterol levels (**D**) and serum triglycerides levels (**E**). Serum interleukin-1β levels (**F**) or interleukin-6 levels (**G**) were measured with kits. All data are expressed as mean ± SD.

The effect of chronic SCH administration on glucose homeostasis and lipid profiles of the HFD mouse model was investigated. Fasting glucose level was significantly elevated in HFD mice, and it was significantly decreased by SCH administration (Fig. 7A). Similarly, serum insulin levels were notably higher in the HFD-fed group but showed significant decreases in both low dose and high dose SCH treated group (Fig. 7B). Calculated with plasma glucose and insulin levels, HOMA-IR index was increased in HFD-fed group but decreased by SCH administration (Fig. 7C). Both serum cholesterol and triglycerides levels were elevated by HFD feeding, yet markedly decreased by SCH intake (Fig. 7D,E).

### SCH attenuated inflammation by decreasing serum inflammatory cytokines

Serous inflammatory cytokines including IL-6 and IL-1β were determined by ELISA. Compared to mice on normal diet, HFD mice showed increased IL-1β level but there was no significance due to high standard deviation within the group. SCH administration caused a decline in serum IL-1β, showing significance only in the high dose SCH group (Fig. 7F). Also, serum IL-6 level was significantly increased by HFD feeding but decreased by SCH supplementation in a dose-dependent manner, showing significance at high dose (Fig. 7G).

### SCH protected from liver damage in HFD-fed mice

**Figure 8:**
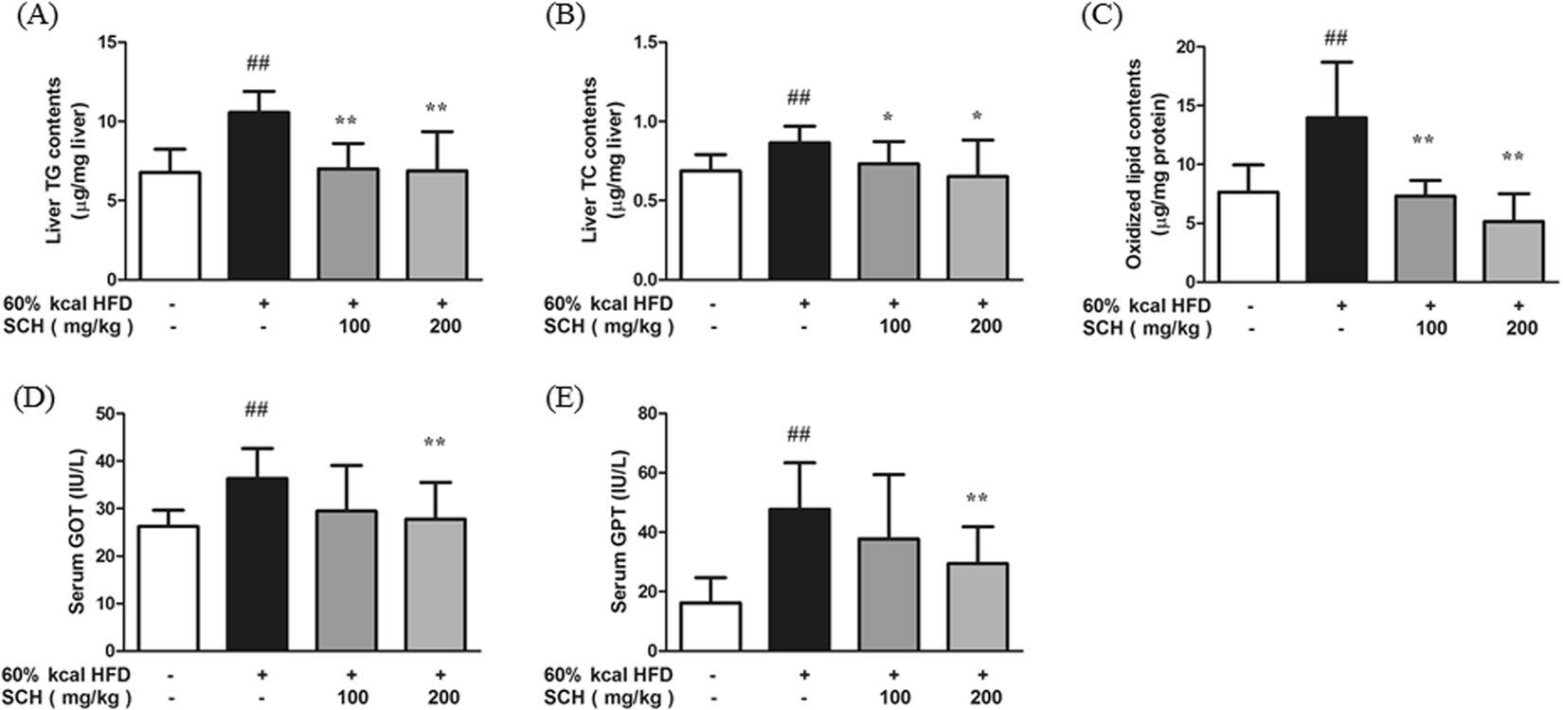
Effect of 15 week SCH administration on hepatic lipid content, and oxidized lipid content in liver and liver enzyme levels of HFD-fed c57BL/6 mice. Hepatic triglycerides (**A**) and total cholesterol (**B**) were measured and normalized to weight of liver analyzed. (**C**) Oxidized lipid content in liver was measured and normalized to protein. Serum GOT (**D**) and GPT (**E**) levels were measured with a kit. All data are expressed as mean ± SD.

The effect of SCH administration on triglycerides and total cholesterol levels and degree of tissue oxidation were assessed in liver. As expected, liver triglycerides and cholesterol levels were increased in HFD group, but significantly decreased by SCH administration at 100, 200 μg/ml concentrations (Fig. 8A,B). Oxidized lipid content of liver tissue exhibited a similar trend (Fig. 8C). Liver enzyme (GOT, GPT) levels in serum were significantly elevated in HFD group, but significantly decreased in high dose SCH group (Fig. 8D,E).

### SCH attenuated hepatic steatosis and hypertrophy of adipose tissue

**Figure 9:**
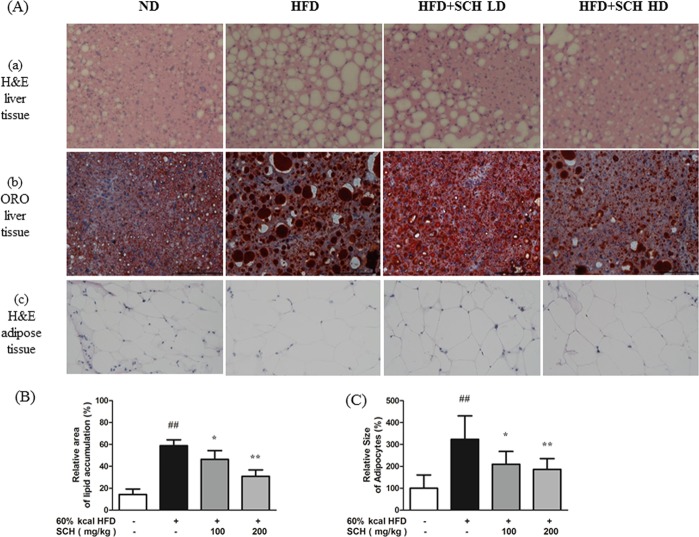
Histologic analysis of tissues showing effects of 15 week SCH administration on hepatic lipid accumulation and size of epididymal adipocytes of HFD-fed c57BL/6 mice. (**A**) Representative images of H&E stained liver tissue (a), Oil Red O stained liver tissue (b), and H&E stained adipose tissue (c) from each group. Quantification of lipid stained area of liver tissue (**B**) and average size of adipocytes (**C**). Data are expressed as mean ± SD.

Histologic analysis of mouse liver tissue was performed using H&E and Oil Red O staining. H&E staining of liver tissue sections revealed excessive accumulation of lipids demonstrated by unstained fat vacuoles (Fig. 9A(a)) and this finding was supported by Oil Red O staining (Fig. 9A(b)). When mice were co-treated with HFD and SCH, histology showed noticeable reduction in lipid vacuoles and stained area (Fig. 9A(a),(b),B).

H&E staining of mice epididymal adipose tissue revealed extensively enlarged adipocytes in HFD group (Fig. 9A(c)). However, adipocyte size was decreased by long-term SCH administration in SCH groups (Fig. 9A (c)). The difference between groups was evident when images were analyzed with size of adipocytes, demonstrating significant reductions in SCH groups as compared to HFD groups (Fig. 9C).

## Discussion

Here we illuminate the feasibility of a new approach using literature-based repositioning of a drug that originated from alternative medicine. In this case, two medical texts written in different eras of Chinese history, suggested different indications for the same herbal composition of SCH. As the indications of formula written in classic works of TCM are simple^28^, with different interpretations by medical practitioners, a herbal prescription can be used for varying indications. Meanwhile, those indications can be changed by references (classics of TCM) which are reflecting the development of medical theory. Also, recent observations that herbal compositions commonly act on multiple targets or multiple symptoms has raised interest in drug repurposing^29^. Therefore, it is worthwhile to revisit the value of ignored or buried indications of herbal prescription in old TCM classics and this approach may be able to provide us with possibilities for redevelopment as a new therapeutic by drug repurposing.

Recent evidence supports functional involvement between liver steatosis and metabolic syndrome, linked by the pathogenic state of insulin resistance^30^. This state is manifested by high basal glucose level^31^, another symptom of MetS, which is the outcome of decreased utilization of blood glucose by tissues^32^. Impaired glucose utilization causes several defects in metabolic aspects of cells^33–35^, and there is a need to investigate new agents to attenuate insulin resistance for treating MetS.

AMPK is a highly-conserved energy sensor and modulator, which regulates energy homeostasis at the cellular and entire body levels^36^. Activated by phosphorylation at Thr172 of α-subunit, AMPK switches from anabolic state to catabolic state^37^, in consequence of which it exerts therapeutic effects against MetS^38^, placing it as a central regulator for MetS investigation. To date, several downstream effectors of AMPK have been heavily studied, because of its ubiquitous expression^39^ and widespread effects on gluconeogenesis, glycolysis lipogenesis, lipolysis etc.^40^. In this study, the attenuating mechanisms of SCH on MetS were investigated, with AMPK and its downstream substrates as central modulators of interest.

As presented above, SCH attenuated glucose metabolism and rescued liver, which is the essential organ for controlling glucose homeostasis, from excessive lipid accumulation *in vitro* and *in vivo*^41^. Primarily by activating AMPK and its downstream signaling in liver, SCH has pleiotropic effects which are beneficial for attenuating hepatic steatosis covering glucose metabolism, adipogenesis, etc. Moreover, immunofluorescence images demonstrated facilitated glucose utilization and potentiated insulin sensitivity by SCH in the FFA-induced hepatic steatosis model. Accordingly, the *in vivo* model showed favorable impacts of SCH administration against HFD-induced MetS with attenuated glucose and lipid profiles in serum. As we expected, SCH administration protected mice liver from lipid overload demonstrated by histologic examination. Results of real-time PCR array unearthed potential genes influenced by SCH treatment in HepG2 cells. Promotion of beta-oxidation by increasing CPT1 level is possibly one of the prominent mechanisms of SCH in liver, as it was demonstrated both by gene expression levels (PCR array) and protein immunoblot analysis (Western blotting).

Adipocyte differentiation has great influence on metabolic profiles of the host since it affects adipocyte maturity thus controlling adipokine production related to obesity^42^. Hypertrophy and hyperplasia of adipocytes is regarded as one of the main causes of obesity^43^. As master regulator genes and transcription factors of adipocyte differentiation, modulating C/EBPα and PPARγ genes are regarded as key to suppressing adipogenesis^44^. As demonstrated by the lipid staining results from 3T3-L1, SCH treatment successfully inhibited lipid accumulation and preadipocyte differentiation, and consequently SCH significantly reduced genes and protein levels associated with adipogenesis. Moreover, staining of mouse adipose tissue revealed that SCH treatment inhibited hypertrophy of adipocytes as determined by average size of adipocytes.

Based on previous studies, each constituent of SCH has potentially therapeutic effects against MetS. *Pharbitis Semen* possesses hypolipidemic effects on liver and serum of rats chronically exposed to ethanol^45^. A water extract of *Cyperi rhizoma* also has attenuating effects on obese rats induced by atherogenic diet including high cholesterol and cholic acid^46^.

As a digestive agent, SCH is expected to exert various impacts on MetS through unrevealed effects on the intestinal tract. Anthocyanins from *Pharbitis nil* has inhibitory effects on intestinal α-glucosidase, which can alleviate hyperglycemia after starch-rich meals^47^. SCH might have invigorated intestinal function and movement, which was not investigated in this work. It is recommended to prescribe bile acid sequestrants to patients with cholesterolemia^48^ and the constituents of SCH are reported to have compounds (teriterpenoid saponins from *Pharbitis nil*) which act as natural bile acid sequestrants^49^.

Unexpectedly however, SCH administration did not significantly reduce body weight in either low dose or high dose SCH groups. The large standard deviation of body weight in both SCH groups implies weight-decreasing mechanisms may be distinctly influenced within each individual mouse. It raises the necessity of additional study with *in vivo* models in order to seek reasonable explanations.

As our data indicate, SCH has the potential to attenuate MetS by enhancing glucose metabolism and inhibiting lipogenesis through activating AMPK and downstream signaling. Identification of active compounds contained in SCH is necessary to fully understand the effectiveness of SCH against MetS that was demonstrated in this study.

## Methods

### Herbal formula preparation

The SCH used in this study was prepared by extracting mixed herbs (*Pharbitis Semen*: *Trogopterorum Faeces*: *Cyperi Rhizoma* = 2: 1: 1, w/w) with reflux extraction method. These herbs were purchased from Humanherb (Gyeongsangbuk-do, South Korea). The hot water extract was filtered and evaporated with rotary evaporator (Buchi, Switzerland) at 95 °C and freeze-dried to obtain final SCH sample (Yield = 15.53%). The SCH was eluted with DPBS and filtered through 0.22 μm syringe filter. Eluted samples were kept at −20 °C before use.

### Cell Culture and Differentiation

HepG2 cells (#88065) and 3T3-L1 cells (#10092.1) were purchased from Korea Cell Line Bank (KCLB). HepG2 cells and 3T3-L1 cells were cultured in Dulbecco’s Modified Eagle Medium (DMEM, Hyclone, South Logan, UT, USA) supplemented with 10% fetal bovine serum (FBS, Gibco, Carlsbad, CA, USA), 100 U/mL penicillin and streptomycin (Gibco, USA). Cell lines were grown at 37 °C under air containing 5% CO_2_ in an incubator with humidified atmosphere. Cells were grown and maintained to approximately 70–80% confluence.

HepG2 cells were seeded on 6 well plates with density of 5 × 10^5^ cells per well and incubated for 24 h. Subsequently, cells were subjected to FBS starvation by incubating in DMEM. Culture medium was replaced with 1% bovine serum albumin (BSA) supplemented DMEM. Mixed free fatty acids were added (1 mM mixture of oleic acid: palmitic acid, 2: 1) into the culture medium for 24 h and washed twice with DPBS. Cells were incubated in DMEM with SCH treatment for 24 h (for Western blotting) or 48 h (for Oil Red O staining).

3T3-L1 cells were seeded on 6 well plates with density of 5 × 10^5^ cells per well and incubated until confluence reached 100%. Cells were maintained at their maximum confluence for 48 h, and media was changed into differentiation media (FBS supplemented DMEM containing 1 μM dexamethasone, 0.5 mM 3-isobutyl-1-methylxanthine, 10 μg/ml insulin) for 72 h. Cells were incubated with maturation media (FBS supplemented DMEM containing 10 μg/ml insulin) for 4 days (for real-time PCR) or 13 days (for ORO staining and Western blotting). Maturation media was replaced with fresh media every 48 h of incubation. SCH was added repeatedly every 48 h with maturation media (for ORO staining and Western blotting) or single-treated at final 12 h before harvest (for real-time PCR).

### Determination of intracellular lipid accumulation in HepG2 cells and 3T3-L1 cells by Oil Red O staining

HepG2 cells and 3T3-L1 cells were washed with DPBS and then fixed with 10% formalin for 1 h at room temperature. After fixation, cells were washed once with 60% isopropanol and dried. Stock solution of Oil Red O staining was prepared by dissolving 0.7 g of Oil Red O powder in 200 ml of isopropanol stirred overnight and then filtered. Stock solution was diluted with distilled water at a ratio of 3: 2, mixed for 20 min and filtered to make working solution. Cells were stained with working solution of Oil Red O for 15 min. Stained cells were washed with distilled water and air-dried. Stained cells were examined under an inverted microscope system with camera (DMI 6000, Leica, Wetzlar, Germany) and stains were re-dissolved in pure isopropanol to measure OD at 520 nm wavelength.

### Determination of intracellular lipid accumulation in HepG2 cells by triglycerides and total cholesterol kit

After 48 h incubation with SCH, HepG2 cells were scraped and transferred to microtubes and centrifuged. Cell pellets were briefly sonicated and lysed. Content of intracellular triglycerides and total cholesterol were determined using commercial colorimetric assay kit (Asan Pharmaceutical, Seoul, South Korea) with provided standards according to manufacturer’s protocol. Results were normalized to protein concentration analysed with commercial BCA protein assay kit (Thermo Fisher Scientific, USA).

### Fluorescence-labeled glucose uptake assay

HepG2 cells were seeded on 96 well black plates with clear bottoms at a concentration of 1 × 10^4^ cells per well. Cells were serum-starved for 24 h and media changed to serum-free 1% BSA DMEM with palmitic acid (250 μM) followed by SCH co-treatment for 24 h. HepG2 cells were washed gently with DPBS and incubated with glucose/serum free DMEM containing 150 μg/ml of 2-NBDG and incubated at 37 °C, in the dark, for 20 min. Unincorporated 2-NBDG was removed by washing with DPBS. Relative fluorescence of cells were measured with fluorescence spectrophotometer (SpectraMax Gemini EM microplate reader, Molecular Devices, USA) at excitation/emission = 485/545. Fluorescent images of 2-NBDG were taken under inverted microscope/camera system equipped with 3 epi-fluorescence filter cube (Eclipse Ts2-FL, Nikon, Tokyo, Japan).

### Fluorescence imaging of FITC-labeled GLUT2 in HepG2 cells

HepG2 cells were seeded on 2-well chamber slides (Thermo Fisher Scientific, USA) and incubated for 24 h. Cells were serum-starved for 24 h and media changed to 1% BSA supplemented DMEM with 1 mM FFA (OA:PA = 2:1, v/v). Cells were incubated with insulin for 30 min and washed and fixed with 4% paraformaldehyde for 10 min at RT. Washed with PBST (0.1% Tween20 in DPBS), cells were blocked with 2% BSA in PBST for 1 h. Chamber slide was incubated overnight with rabbit GLUT2 antibody (1:500, 1% BSA in PBST, Santa Cruz, USA). Cells were rewashed with PBST and incubated with FITC-conjugated Alexa Fluor 488 goat anti-rabbit IgG antibody (1:500, 1% BSA in PBST, Thermo Fisher Scientific, USA) for 2 h in dark. Cells were washed to detach incorporated antibodies and mounted with antifade DAPI mounting medium (Vector Laboratories, Peterborough, UK). Slides were examined under a fluorescence microscope system with camera.

### Western blotting

HepG2 cells and 3T3-L1 cells were treated as described above before harvest. Cells were washed with ice-cold DPBS and lysed with radioimmunoprecipitation assay buffer (RIPA, Thermo Fisher scientific, USA) containing protease inhibitor and phosphatase inhibitor cocktail (Gendepot, Barker, TX, USA). Protein concentration was measured using BCA kit (Thermo Fisher Scientific, USA). Thirty micrograms of whole protein lysate with protein loading buffer were loaded on 10% SDS-PAGE gels and electrophoresed. Protein was transferred to PVDF membrane at 100 V for 90 min using Mini-transblot electrophoretic transfer cell (Bio-rad, Hercules, CA, USA). Membranes were blocked with 5% BSA in TBST for 1 h. Membranes were further incubated with primary antibodies (diluted in 3% BSA in TBST, 1:1000) at 4 °C overnight with gentle shaking and washed. Membranes were incubated with secondary antibodies (diluted in 1% BSA in TBST, 1:2000) for 2 h and washed. Immunoreactive bands were detected with western blot imaging system (Fusion Solo, Vilber Lourmat, Collégien, France). Immunoblots were detected using chemiluminescent ECL buffer (SuperSignal West Pico, Thermo Fisher Scientific, USA)

### Quantitative real-time PCR

Following the treatment described above, total RNA was isolated from 3T3-L1 cells using Trizol reagent (Thermo Fisher Scientific, USA) according to instructions. Quantity and integrity of RNA was checked for assuring quality of samples. Reverse transcription was performed by using AccuPower RT PreMix (Bioneer, Daejeon, South Korea) and oligo (dt) 18 primers (Invitrogen, Carlsbad, CA, USA). Amplification of primer-specific binding cDNA was performed using LightCycler 480 PCR system (Roche, Basel, Switzerland). Reactants include 10 μl of 2x SYBR green Master mix (Roche, Switzerland), 8 μl of ultrapure water, 10 pmol/μl of primers and 1 μl of template cDNA. Amplication process consists of first denaturation at 95 °C for 10 min, following 45 cycles of denaturation at 95 °C for 10 s, annealing at 50~56 °C for 20 s and extension at 72 °C for 20 s; and melting curve analysis at 95 °Cfor 5 min. Threshold cycle (Ct value) was calculated to quantify the PCR product. The relative result was calculated by dividing the Ct result of specific gene with Ct result of β-actin gene for normalization. All data were acquired with LightCycler 480 instrument and software. Primers used in this study are listed in Table S1.

### Animals

Twenty four, 4-week old male C57BL/6J mice was purchased from Orientbio (Gyeonggi-do, South Korea). Mice were housed in a controlled environment of temperature (25 °C), humidity (50%) and 12 h dark/light cycle with free access to diet and water. After 1 week of acclimatization, mice were randomly divided into four groups and maintained for 15 weeks; Normal diet group fed AIN93G diet (ND, n = 6), high fat diet group fed 60% high fat diet (HFD, n = 6), high fat diet with low dose SCH group (LD, 100 mg/kg/day, n = 6), high fat diet with high dose SCH group (HD, 200 mg/kg/day, n = 6). All diets were purchased from Saeronbio (Gyoenggi-do, South Korea). The SCH was eluted with distilled water and administered daily by oral administration with gavage. Bodyweight and food intake was checked weekly. After 15 weeks of administration, all mice were subjected to oral glucose tolerance test (OGTT). After designated periods, mice were sacrificed for collecting serum and organs. Organ pieces were stored in RNA later (Invitrogen, USA) and frozen at −80 °C for further analysis. Sera were separated by centrifuging blood. All protocols for animal experiments were approved by Dongguk University Ethics Committee (Approval No. IACUC-2016-055-1) and were carried out in accordance with the approved guidelines.

### Oral glucose tolerance test (OGTT)

One week before sacrifice, all mice were starved for 12 h. Before glucose challenge, fasting glucose level was checked using blood drops from tail. Subsequently, all mice were administered glucose solution (200 mg/ml) of 2 g/kg bodyweight. Blood glucose levels were determined at 30, 60, 90, 120 min using ACCU-CHEK glucose meter (Roche Applied Science, Basel, Switzerland). HOMA-IR as indexes of insulin resistance was calculated using the formula:

$$\mathrm{HOMA} \mbox{-} \mathrm{IR}={\rm{Fasting}}\,{\rm{glucose}}\times {\rm{Fasting}}\,{\rm{insulin}}(\mathrm{mU}/{\rm{L}})/22.5$$

### Serum analysis

Mice sera were isolated by centrifuging whole blood at 3000 rpm for 20 min. Serum triglycerides (TG), and total cholesterol (TC) were measured using a colorimetric kit (Asan Pharmaceutical, South Korea) with provided standards. Assay for serum hepatic enzyme was performed using oxaloacetic transaminase/glutamic pyruvic transaminase (GOT/GPT) test kit (Asan Pharmaceutical, South Korea). Serum insulin, serum interleukin-6 and interleukin-1β levels were determined using mouse insulin (Mercodia AB, Uppsala, Sweden), IL-6, IL-1β (Thermo Fisher Scientific, USA) ELISA kit following manufacturer’s instructions.

### Hepatic lipid content analysis

Part of mouse liver piece was weighed and homogenised for total lipid extraction using chloroform and methanol (2:1, w/w). Fractionated lipid was air-dried and eluted with pure isopropanol. Total cholesterol and triglycerides were determined using TC, TG kit (Asan Pharmaceutal, South Korea).

### Lipid peroxidation measurement

The same weight of mouse liver was homogenised (mixed with 1.15% KCl buffer, 1:9, w/w) from each individual to measure lipid peroxidation levels. Liver homogenate was mixed with 2-thiobarbituric acid (TBA, 0.81%, w/w) and incubated at 95 °C for 1 h to form TBA-chromogen. The reaction was stopped by cooling, and 1 ml of distilled water was added and vortexed. A mixture of pyridine and butanol (5 ml, pyridine: butanol = 1:15) was added to the reactant and centrifuged at 3000 rpm for 30 min. Supernatant of each reactant was transferred to 96 well plates and absorbance was read at 532 nm. Varying concentrations of 1,1,3,3-tetraethoxy propane served as standard.

### Histology

For histologic examination, liver and adipose tissues of mice were embedded in frozen section media (FSC 22, Leica, Jena, Germany) and frozen at −20 °C (liver), or −25 °C (adipose tissue). Specimens were prepared by cutting samples into 8 μm thickness using CM 1860 cryomicrotome system (Leica, Jena, Germany). Specimen slides (Marienfeld, Lauda-Königshofen, Germany) was dried and fixed in 10% formalin solution for 5 min. Liver sections were stained with Oil Red O (dissolved in propylene glycol) for 30 min and Mayer’s Hematoxylin as counterstain for 1 min. Liver and adipose tissue were stained with Hematoxylin & Eosin to examine structural changes. Stained slides were examined under microscope (DMI 6000, Leica, Germany) equipped with DFC480 camera (Leica, Germany).

### Statistical analysis

All data are expressed as mean ± standard deviation (SD). All data were subjected to statistical analysis using Graph Pad Prism 5.0 and SigmaStat (version 3.5, Systat Software, CA, USA). Statistical significance was assessed by comparison with control group using Student’s *t*-test with Bonferroni post hoc test. The differences were considered as statistically significant at p < 0.05. For drawing correlation curve of content with standard attributes to MS excel. Hierarchical clustering heatmap analysis of PCR array result was conducted using RT^2^ Profiler PCR Array data analysis version 3.5.

## Supplementary information

Supplementary info
