# Supplementary material for: Drug repurposing in alternative medicine: herbal digestive Sochehwan exerts multifaceted effects against metabolic syndrome
Source: Sci Rep. 2019 Jun 21;9:9055. doi: 10.1038/s41598-019-45099-x (PMC6588698; doi:10.1038/s41598-019-45099-x)
Supplement: Supplementary file 1 — Supplementary info [file 41598_2019_45099_MOESM1_ESM.pdf]

`

# Drug repurposing in alternative medicine: herbal digestive Sochehwan exerts multifaceted effects against metabolic syndrome

Dong-Woo Lim[1], Hyuck Kim[1], Young-Mi Kim[2], Young-Won Chin[2], Won-Hwan Park[1], Jai-Eun Kim[1]*

1 College of Korean Medicine, Dongguk University-Gyongju, Gyeonggi-do 10326, Republic of Korea

2 College of Pharmacy and Integrated Research Institute for Drug Development, Dongguk University-Seoul, Gyeonggi-do 10326, Republic of Korea

Corresponding author

Jai-Eun Kim

Electronic address: herbqueen@dongguk.ac.kr

`

# Supplementary methods

## Chemicals and reagents

For in vitro study, Oil-Red O, oleic acid (OA), palmitic acid (PA), 3-isobutyl-1-methylxanthine (IBMX), and dexamethasone were all purchased from Sigma Chemicals (St. Louis, MO, USA). 2-NBDG (2-(2-(N-(7-Nitrobenz-2-oxa-1,3-diazol-4-yl)Amino)-2-Deoxyglucose)) was purchased from Thermo Fisher Scientific (Waltham, MA, USA). Antibodies to phosphorylated acetyl-CoA carboxylase (p-ACC), acetyl-CoA carboxylase (ACC), phosphorylated 5' AMP-activated protein kinase (p-AMPK), 5' AMP-activated protein kinase (AMPK), CCAAT/Enhancer Binding Protein (C/EBP), and carnitine palmitoyltransferase 1 (CPT1) were purchased from Cell Signaling Technology (Beverly, MA, USA). Peroxisome proliferator-activated receptor gamma (PPAR-γ) antibodies were purchased from Abcam (Cambridge, UK). Leptin, GLUT2, β-actin antibodies were purchased from Santa Cruz (Santa Cruz, California, USA). HRP-conjugated secondary antibodies were purchased from Santa Cruz (USA). Alexa fluor 488 conjugated goat anti-rabbit antibody was purchased from Thermo Fisher Scientific (USA). Oligo nucleotide primers used in real-time PCR were purchased from Macrogen (Seoul, South Korea).

## Determination of cell viability

Effect of SCH on cell viability was determined by EZ-Cytox cell viability assay kit (Daeil Lab Service, Seoul, Korea) following the kit protocol. HepG2 cells and 3T3-L1 cells were seeded at 96 well plates with density of $1x10^4$ cells per well and incubated for 24 h. Cells were incubated with various concentrations (0-100 μg/ml) of SCH for 24 h. EZ-Cytox kit reagent was added to each well and then incubated at 37℃ for 2 h. Optical density of supernatant was measured at a wavelength of 450 nm using microplate spectrophotometer (VersaMax, Molecular Devices, Sunnyvale, CA, USA)

`

## Western blotting for insulin signaling proteins

Steatosis of HepG2 cells were induced by conditions described in manuscript (1 mM, OA:PA = 2:1, for 24 h). Steatotic HepG2 cells were washed twice with DPBS before changing culture medium. Cells were further incubated in fresh DMEM with SCH for 24 h. Human recombinant insulin was added to culture medium (50 nmol) for 30 min. Cells were scraped and lysed with RIPA (Thermo Fisher Scientific, USA) containing protease inhibitor and phosphate inhibitor (Gendepot, USA). Whole protein was subjected to Western blotting with same protocol as described in Methods section.

## Gene expression profiling by RT$^2$ PCR array

Steatosis of HepG2 cells were induced by conditions described in ‘Cell culture and differentiation’ section of Methods (1 mM, OA:PA = 2:1, for 24 h). Intact RNA was extracted from steatotic HepG2 cells after 3 h of SCH treatment and reverse transcription was performed for preparation of quantitative real-time PCR array. Genes involved in Human liver steatosis were investigated by Human Fatty Liver RT$^2$ Profiler PCR Array kit (PAHS-157Z, Qiagen, Hilden, Germany) according to manufacturer's protocol. All data acquired from PCR array were analyzed by following calculation and analysis process on web Data Analysis Center

(http://pcrdataanalysis.sabiosciences.com/pcr/arrayanalysis.php)

`

## Supplementary Figure and Table

| Parameters | Animal Groups | | | |
|---|---|---|---|---|
| | ND | HFD | HFD+SCH LD (100 mg/kg) | HFD+SCH HD (200 mg/kg) |
| 0 week B.W (g) | 18.75 ± 1.40 | 18.70 ± 0.92 | 18.43 ± 1.49 | 18.82 ± 1.01 |
| 15 week B.W (g) | 28.02 ± 2.99 | 42.70 ± 2.92# | 38.85 ± 10.54 | 39.93 ± 8.26 |
| FER per group (0~15 week) | 0.040 | 0.110 | 0.096 | 0.094 |
| Liver weight (g) | 1.00 ± 0.11 | 2.13 ± 0.33# | 1.85 ±1.04 | 1.82 ± 0.52 |
| Intestine fat weight (g) | 0.34 ± 0.11 | 1.15 ± 0.46# | 0.97 ± 0.68 | 1.12 ± 0.53 |
| Gonadal fat weight (g) | 1.02 ± 0.31 | 2.62 ± 0.29# | 2.15 ± 0.89 | 2.25 ± 0.51 |
| Renal fat weight (g) | 0.26 ± 0.09 | 0.93 ± 0.14# | 0.78 ± 0.34 | 0.90 ± 0.26 |

**Table S1. Effect of 15 week SCH administration on body weight, food efficiency ratio (FER) and organ weight in high fat diet-fed mice. All data are expressed as mean±SD. # shows statistically significant difference at p<0.05 as compared with the ND group. * shows statistically significant difference at p<0.05 as compared to the HFD group**

`

| Primer | Sequence | |
|---|---|---|
| | Sequence of Forward Primer (5'-3') | Sequence of Reverse Primer (5'-3') |
| C/EBPα | GCGCAAGAGCCGAGATAAAG (20 mer) | CACGGCTCAGCTGTTCCA (18 mer) |
| PPARγ | ATGCCAAAAATATCCCTGGTTTC (23 mer) | GGAGGCCAGCATGGTGTAGA (20 mer) |
| SCD1 | ATATCCTGGTTTCCCTGGGT (20 mer) | CAGCGGTACTCACTGGC (17 mer) |
| SREBF | GGAACAGACACTGGCCGA (18 mer) | AAGTCACTGTCTTGGTTGTTGAT (23 mer) |
| FABP4 | CAGAAGTGGGATGGAAAGTCG (21 mer) | CGACTGACTATTGTAGTGTTTGA (23 mer) |
| ACC1 | TGGCGTCCGCTCTGTGATA (19 mer) | CATGGCGACTTCTGGGTTG (19 mer) |
| β-Actin | GACGGCCAGGTCATCACTATTG (22 mer) | CCACAGGATTCCATACCCAAGA (22 mer) |

**Table S2 Primers used in quantitative real-time PCR**

`

(A) (B)

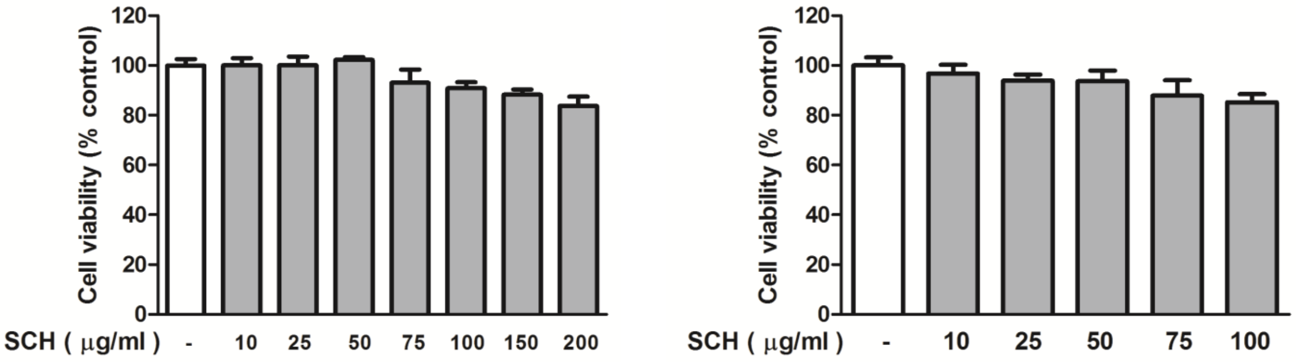


**Fig. S1 Impact of SCH treatment on (A) 3T3-L1 cell viability and (B) HepG2 cell viability. Results are expressed as percentage of non-treated control. All data are expressed as mean±SD.**

`

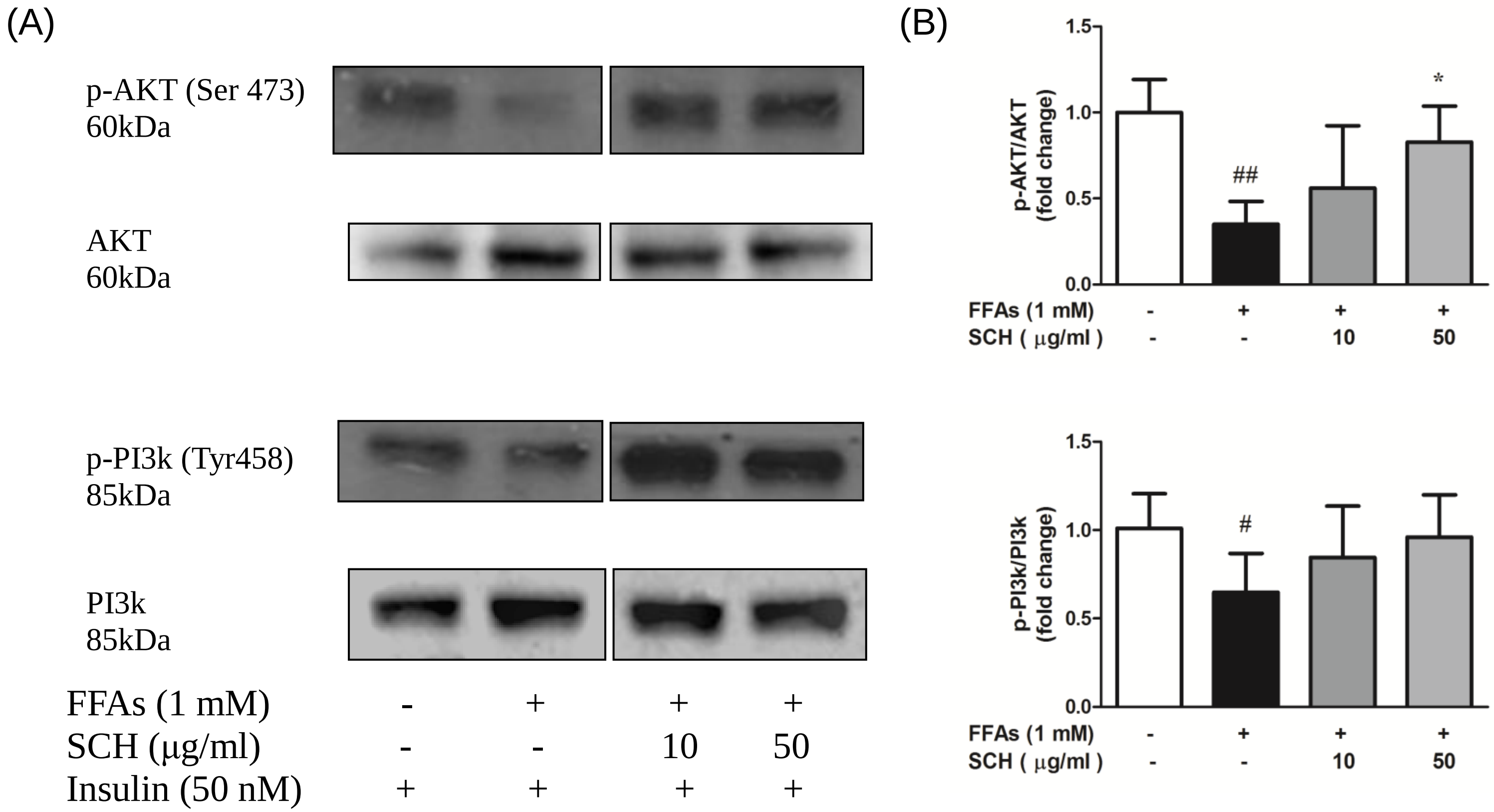


**Fig. S2 Western blot analysis showing the effect of SCH on phosphorylation of proteins involved with insulin signaling pathway after insulin stimulation in FFA-induced steatotic HepG2 cells. (A) Representative blots of proteins are depicted. (B) Band intensity was measured with densitometric analysis and normalized to the intensity of non-phosphorylated AKT or PI3k. Data are expressed as mean±SD.**

**Full-length blots are represented in Supplementary Figure 4.**

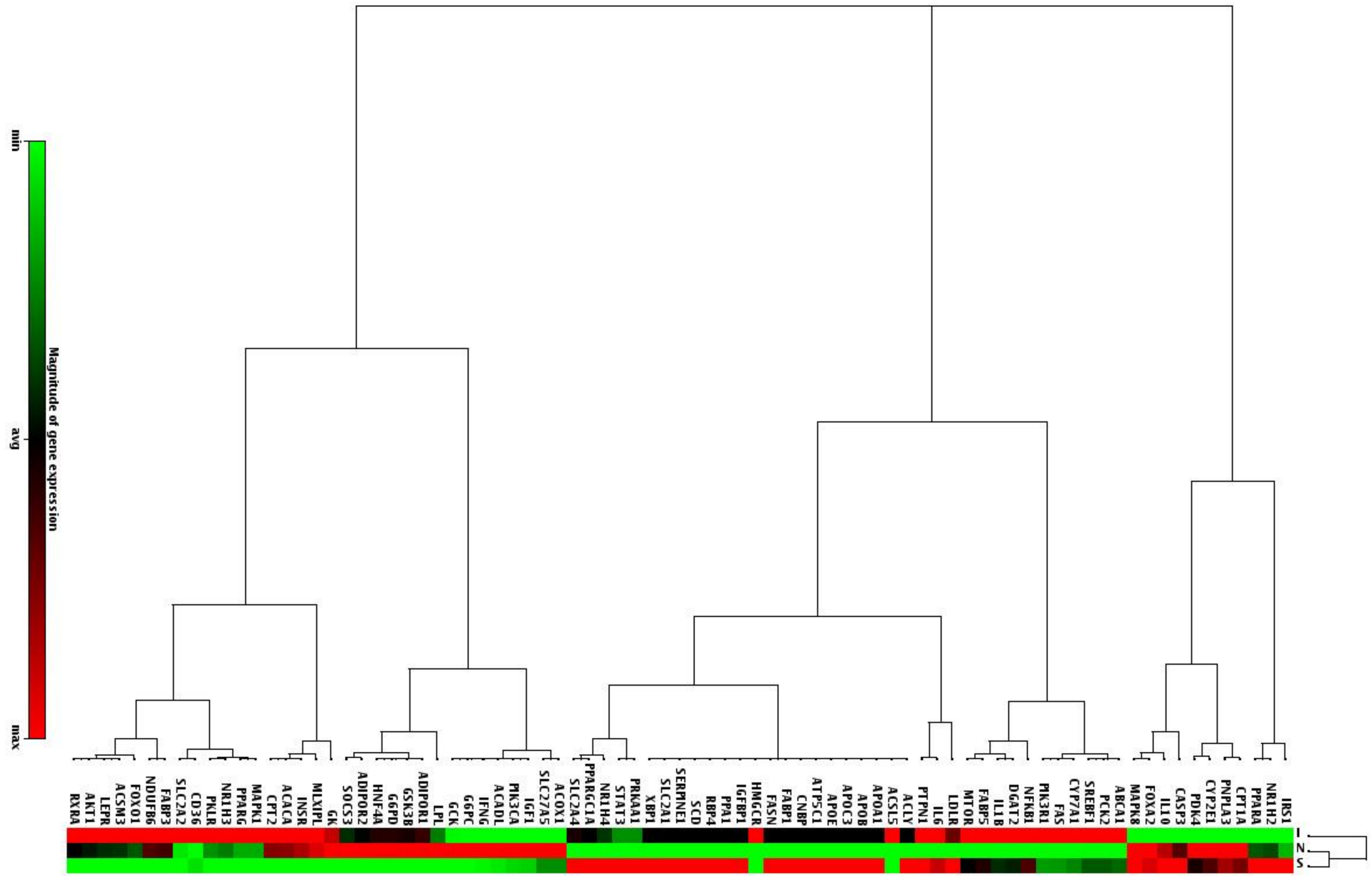

min
avg
max
Magnitude of gene expression
RXRA
AKT1
LEPR
ACSM3
FOXO1
NDUFB6
FABP3
SLC2A2
CD36
PKLR
NR1H3
PPARG
MAPK1
CPT2
ACACA
INSR
MLXIPL
GK
SOCS3
ADIPOR2
HNF4A
G6PD
GSK3B
ADIPOR1
LPL
GCK
G6PC
IFNG
ACADL
PIK3CA
IGF1
SLC27A5
ACOX1
SLC2A4
PPARGC1A
NR1H4
STAT3
PRKAA1
XBP1
SLC2A1
SERPINE1
SCD
RBP4
PPA1
IGFBP1
HMGCR
FASN
FABP1
CNBP
ATP5C1
APOE
APOC3
APOB
APOA1
ACSL5
ACLY
PTPN1
IL6
LDLR
MTOR
FABP5
IL1B
DGAT2
NFKB1
PIK3R1
FAS
CYP7A1
SREBF1
PCK2
ABCA1
MAPK8
FOXA2
IL10
CASP3
PDK4
CYP2E1
PNPLA3
CPT1A
PPARA
NR1H2
IRS1
I
N
S

`

**Fig. S3 Hierarchical clustering result of PCR array of genes associated with human hepatic steatosis in HepG2 cells. The heatmap diagram denotes the level of gene expression of each group normalized to that of the FFA-induced group. Dendrogram height represents relative measure of dissimilarity between clusters. N : control group, I : FFAs-induced steatosis group, S : exposed to FFAs with SCH group.**

`

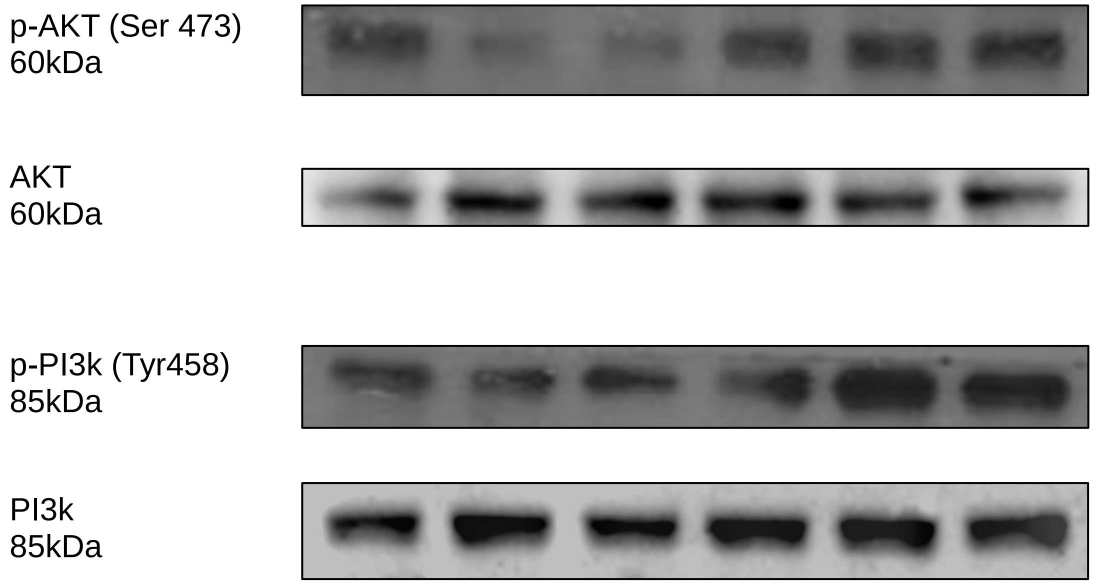


**Fig. S4 Full-length blots of Supplementary Figure 2. Proteins from HepG2 Cells treated with other test samples were loaded at Lane 3 and Lane 4.**
